# Quantitative Magnetic Resonance Imaging of Cortical Multiple Sclerosis Pathology

**DOI:** 10.1155/2012/742018

**Published:** 2012-11-18

**Authors:** Christine L. Tardif, Barry J. Bedell, Simon F. Eskildsen, D. Louis Collins, G. Bruce Pike

**Affiliations:** ^1^McConnell Brain Imaging Centre, Montreal Neurological Institute, Montreal, Canada; ^2^Small Animal Imaging Laboratory, McConnell Brain Imaging Centre, Montreal Neurological Institute, Montreal, Canada; ^3^Center of Functionally Integrative Neuroscience, Aarhus University, Aarhus, Denmark

## Abstract

Although significant improvements have been made regarding the visualization and characterization of cortical multiple sclerosis (MS) lesions using magnetic resonance imaging (MRI), cortical lesions (CL) continue to be under-detected *in vivo*, and we have a limited understanding of the causes of GM pathology. The objective of this study was to characterize the MRI signature of CLs to help interpret the changes seen *in vivo* and elucidate the factors limiting their visualization. A quantitative 3D high-resolution (350 **μ**m isotropic) MRI study at 3 Tesla of a fixed *post mortem* cerebral hemisphere from a patient with MS is presented in combination with matched immunohistochemistry. Type III subpial lesions are characterized by an increase in T1, T2 and M0, and a decrease in MTR in comparison to the normal appearing cortex (NAC). All quantitative MR parameters were associated with cortical GM myelin content, while T1 showed the strongest correlation. The histogram analysis showed extensive overlap between CL and NAC for all MR parameters and myelin content. This is due to the poor contrast in myelin content between CL and NAC in comparison to the variability in myelo-architecture throughout the healthy cortex. This latter comparison is highlighted by the representation of T1 times on cortical surfaces at several laminar depths.

## 1. Introduction

Although multiple sclerosis (MS) was until recently thought of as primarily a white matter (WM) disease, the involvement of grey matter (GM) was reported as far back as the beginning of the 20th century [1]. The interest in GM pathology was likely repressed due to the lack of prominence in conventional histopathology and magnetic resonance imaging (MRI). However, over the last decade, pathological investigations using immunohistochemistry (IHC) staining methods against myelin antigens have revealed the full extent of cortical pathology [2]. 

 The pathological hallmark of cortical lesions (CLs) is demyelination. They also occasionally exhibit a minor microglial reaction, axonal transection, as well as neuronal, glial, and synaptic loss [3–5]. Bø et al. defined a system of CL classification that distinguishes mixed GM-WM lesions (type I) from purely GM lesions [2]. The latter include small intracortical lesions (type II), subpial lesions that affect the superficial cortical layers and may extend over several gyri (type III), and lesions affecting the entire width of the cortex from pial surface to the subcortical WM (type IV). Type III subpial lesions are most extensive and frequent, and may lead to general subpial demyelination affecting up to 70% of the total cortical area [2, 6]. Cortical damage has been observed in the early relapsing-remitting stage [7], but is more characteristic of the progressive phase of MS [6]. 

The lack of sensitivity of MRI to CLs is in part due to the different pathophysiology of GM lesions in comparison to those of WM, in particular the reduced amount of inflammation and edema that cause hyperintense WM lesions on T2-weighted images. The contrast between demyelinated CLs and surrounding normal appearing cortex (NAC) is further reduced due to the lower myelin content of GM, about 10% that of WM, particularly in the superficial laminae. Visibility of CLs on MR images is also hindered by their small size. Subpial lesions may cover quite a large cortical area; however, their morphology causes them to be concealed by partial volume effects with adjacent cerebrospinal fluid. The long T1 times of GM and CLs also limit the signal-to-noise ratio.

Several advanced imaging techniques have improved the visualization of CLs *in vivo*. One of the most promising techniques thus far is double inversion recovery (DIR), a T2-weighted turbo spin echo based sequence with inversion times adjusted to null WM and cerebrospinal fluid signals. DIR shows a significant increase in CL detection over conventional MRI, including T2-weighted spin echo (T2-SE) and fluid-attenuated inversion recovery (FLAIR) [8–11]. DIR is inherently characterized by a low SNR, and is sensitive to flow and pulsation artifacts that sometimes cause false positives. Multi-contrast acquisition protocols have been proposed where DIR is combined with T1-weighted sequences such as spoiled gradient echo (SPGR) [12], phase sensitive inversion recovery spin echo [13] or magnetization prepared rapid acquisition by gradient echo [14–16] for more sensitive and reliable CL detection and classification. Combined MRI and neuropathology studies of fixed [17, 18] and fresh [19] *post mortem* MS brain tissue have shown that CLs, in particular type III subpial lesions, remain significantly under-detected using these sequences. The subset of CL load detected using DIR is highly correlated with overall CL load and demyelinated area [18], and is associated with disability, especially cognitive impairment [20–22].

Ultra-high field imaging (≥7 Tesla (T)) and multi-channel phased array coils have also contributed to improved *in vivo* imaging of cortical MS pathology. Due to the increase in signal-to-noise ratio and accelerated acquisition, images are acquired at higher resolutions to detect small CLs, in particular thin subpial lesions. The improved lesion conspicuity allows for more reliable classification [16, 23], as well as higher inter-rater agreement [24]. T2∗-weighted contrast is enhanced at ultra-high fields and shows laminar detail within the cortex. T2∗ contrast between CLs and NAC is higher than phase, T1- and T2-weighted contrast at 7 T [25]. 

There have been significant improvements in imaging of CLs *in vivo* over the last 10 years, but we are still only seeing the “tip of the pathological iceberg” [18]. Recently, recommendations have been published on how to manually score CLs on DIR images, yet consensus on the scoring between research groups remains surprisingly low [26]. 

The development of quantitative MRI (qMRI) acquisition and analysis techniques to detect and characterize cortical pathology *in vivo *is important to improve the specificity to pathological substrates, to capture the full extent of the disease, and identify reliable/reproducible quantitative MR markers of disease burden. Very few studies have looked at the qMRI signature of CLs *in vivo *due to the difficulty in visualizing them [27, 28]. Combined *post mortem* MRI and histopathology are necessary to study the association between the qMRI signature of CLs and neuropathological substrates [18, 29].

Two recent *post mortem* MRI and histopathology studies have shown that the main difference between MR-visible and MR-invisible CLs is lesion size [18, 30], and no significant difference was found in qMR parameters and histopathology [18]. Although an increase in resolution improves visualization of cortical lesions, tissue contrast is also a limiting factor [31]. If the CL is small with respect to the voxel size and the contrast between the CL and neighboring normal appearing brain tissue is low, the CL can be concealed by partial volume effects. This is particularly problematic close to the high contrast boundary between GM and cerebrospinal fluid.

This study consists of a combined high-resolution, 3 T qMRI acquisition and quantitative histological investigation of a formalin-fixed, whole *post mortem* cerebral hemisphere from a patient with MS. The MR parameters me asured include T1 and T2 relaxation times, relative proton density (or equilibrium magnetization) M0, and magnetization transfer ratio (MTR). Myelin content was quantified in sections immuno-stained for myelin basic protein (MBP). The histological sections were spatially registered to the qMRI maps, and voxel-based statistics were performed to investigate the relationship between qMR parameters and the underlying pathological substrates. In addition, tissue classification and cortical surface extraction was performed on the MR images to visualize the spatial extent of the disease and variability in MR parameters across the cortex.

## 2. Methods

### 2.1. Brain Tissue Sample

The right hemisphere of a clinically diagnosed MS patient was provided by the Douglas Hospital Research Centre Brain Bank. The female patient was diagnosed with MS at 53 years of age and suffered an aggressive disease course. She died at the age of 79 due to aspiration pneumonia caused by severe MS. Her final EDSS score was 10. She was diagnosed with clinical dementia two years prior to death, with a mini mental state examination of 23/30. The patient's right hemisphere was fixed in 10% neutral-buffered formalin after a *post mortem* delay of 41.25 hours, and had been fixed for approximately 4 years. 

 A 1 cm coronal slice of healthy control tissue from a separate fixed brain was provided for comparison by the Department of Neuropathology of the Montreal Neurological Institute.

### 2.2. MRI Acquisition

All images were acquired on a Siemens TIM Trio 3 T whole-body MRI scanner with the body transmit coil and a 32-channel receive-only head coil. The MS hemisphere was placed in an MR-compatible cylindrical container filled with formalin. 3D sagittal images were acquired with 350 *μ*m isotropic resolution, 512 × 512 × 240 matrix size and 3/4 partial Fourier phase encoding. The total acquisition time was of approximately 55 hours.

Relaxometry was performed using the variable flip angle method also known as driven equilibrium single pulse observation of T1 and T2 (DESPOT) [32]. T1 and relative proton density, M0, maps were derived from two SPGR images with a constant echo time (TE) of 3.35 ms and repetition time (TR) of 7.7 ms. The flip angles, optimized for the range of relaxation times of the fixed *post mortem* brain, were 4° and 22°. T2 maps were derived from two balanced steady state free precession (bSSFP) images with a fixed TE of 3.84 ms and TR of 7.7 ms, and optimal flip angles 20° and 70°. Each sequence was acquired with 49 signal averages.

MTR was calculated as the percentage decrease in signal caused by a magnetization transfer saturation pulse ((*M*_0_ − *M*_sat⁡_)/*M*_0_∗100%). Two proton density-weighted SPGR images were acquired with *α*, TE and TR set to 25°, 4.09 ms and 25 ms respectively, with 13 signal averages. The second acquisition included the saturation pulse provided on the Siemens Trio 3 T scanner: a 500° Gaussian pulse of 10 ms and 1200 Hz off-resonance with a 100 Hz bandwidth. 

Due to the significant variation in the radio frequency transmission field at 3 T, we acquired a B1 map [33] to correct the nominal flip angles for both DESPOT techniques. The ∆B1 map was derived from two magnetization-prepared turbo spin echo images with echo spacing and TR set to 15 ms and 2 s respectively, and an echo train length of 7. The acquisition was 2D with 2 mm isotropic resolution and 128 × 128 × 50 matrix size. The readout train was preceded by an *α* and 2*α* pulse, where *α* is 20°, for the first and second acquisition respectively. The magnetization preparation is followed by a time delay equal to half the echo spacing.

After scanning the whole hemisphere, it was cut into 1 cm coronal slices and placed in petri dishes filled with formalin. The dishes were stacked and placed into the 32-channel coil and scanned a second time before neuropathological examination, to facilitate histology-to-MRI registration. The entire 3D imaging protocol described above, including relaxometry MTR and B1 measurements, was acquired with a slice thickness of 1 mm.

### 2.3. MRI Analysis

**Figure 1:**
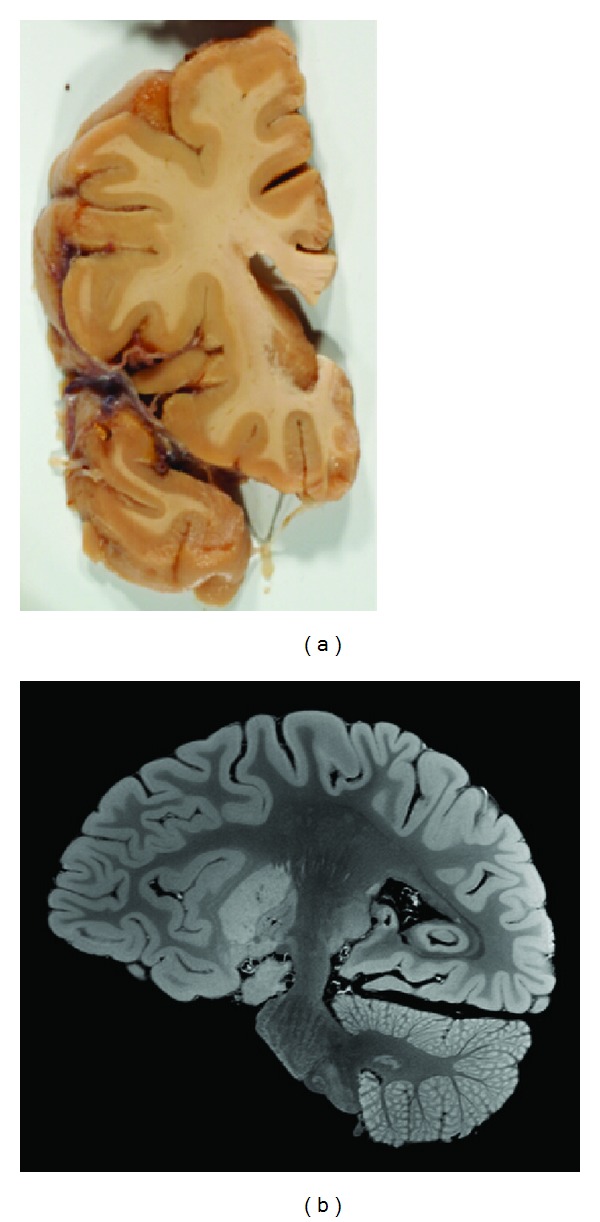
(a) photograph of a coronal slice of the *post mortem* MS hemisphere used for neuropathology. (b) MT-weighted SPGR image of the *post mortem* MS hemisphere at an isotropic resolution of 350 *μ*m.

The MTR map, which most resembles *in vivo* T1-weighted contrast, was used for cortical surface extraction. The MTR image was linearly aligned using 9 degrees of freedom (3 rotation, 3 translation and 3 scale) [34] and then non-linearly warped [35] to the right hemisphere of the ICBM152 non-linear atlas [36] and masked. The non-uniformity corrected MT-weighted SPGR image was used in combination with the MTR map for discrete tissue classification [37]. As shown in Figure 1, the formalin is clearly distinguishable from the surrounding GM in the sulci on the MT-weighted SPGR image due to the high contrast and resolution. The discrete tissue classification results were subsequently corrected for partial volume effects [38] and fuzzy tissue maps were created for WM, GM and formalin. 

Prior to cortical surface segmention, stereotaxic masks were applied to remove the brain stem and cerebellum from the tissue maps, and to label the subcortical GM and ventricles as WM. Small manual corrections of the tissue masks were required due to the deformations caused by the fixation, otherwise the preprocessing was fully automated. An edge map of the pial surface was created by calculating the gradient of the sum of the WM and GM fuzzy tissue maps. The cortical boundary surfaces were extracted using Fast Accurate Cortex Extraction (FACE) [39, 40], which uses deformable surfaces driven by gradient vector fields in a force balancing scheme. 

### 2.4. Neuropathology and Immunohistochemistry

After MRI scanning, the 1 cm thick coronal slices of the formalin-fixed MS hemisphere were photographed. Five tissue blocks of approximately 25 × 25 × 10 mm^3^ were cut from one coronal slice of the MS brain, shown in the photograph of Figure 1. Five blocks were also cut from a matching coronal slice of healthy control brain tissue. The tissue blocks were dehydrated through graded alcohols and xylene, and embedded in paraffin wax. Five 5 *μ*m thick coronal sections, one for each stain, were cut from each block using a rotary microtome, for a total of 50 sections. The sections were separately mounted on positively-charged microscope slides (Superfrost Plus, Fisher Scientific, ON, Canada). The sections from healthy control and MS tissue were handled identically and simultaneously. 

 A section from each block was stained with hematoxylin & eosin, and cresyl violet. The slides were mounted using Permount (Fischer Scientific). All IHC studies were performed using reagents obtained from Thermo Scientific on a Lab Vision Autostainer 360. Tissue sections underwent heat-induced antigen-retrieval in boiling 10 mM citrate buffer (pH 6.0). The following primary antibodies were used: mouse monoclonal antibody against myelin basic protein (MBP, Covance SMI-99P, 1 : 500, 90 min), rabbit antibody against ionized calcium binding adaptor molecule 1 (IBA1, Wako 019–19741, 1 : 200, 90 min) for macrophages and microglia, and mouse monoclonal anti-human glial fibrillary acidic protein (GFAP-Clone 6F2, Dako Cytomation M0761, 1 : 200, 60 min) for astrocytes. All stains were followed by amplification with anti-rabbit biotin-streptavidin-horseradish peroxidase (HRP) and visualization with AEC chromogen. IHC slides were counterstained for 1 minute in 0.1% Acid Blue 129 (Sigma-Aldrich Canada; Oakville, ON, Canada), rinsed for 30 s in sodium acetate-acetic acid buffer (pH 3.6), and air-dried. The slides were mounted in a specially-formulated aqueous mounting media [41]. 

The slides were digitized using a Zeiss MIRAX Scan 150 ultra-high-resolution, automated slide scanner (Carl Zeiss Canada; Toronto, ON, Canada) 48 hours after staining for optimal contrast from the Acid Blue 129 counterstaining. The myelin fibers in the MBP-stained sections were segmented from the background counterstain using an automated image segmentation algorithm based on the high degree of color separation between the red-brown AEC chromogen and the light blue counterstain [41]. The resulting binary maps of myelin fibers were down-sampled in-plane by averaging the pixels to match the resolution of the qMR images, thereby generating a 2D map of myelin content as a percentage of local area.

### 2.5. MRI-Histology Registration

For each MR contrast acquisition, the individual MR image repetitions were linearly aligned prior to averaging to correct for any image drift and small movements of the hemisphere within the container [34]. The average images were used to calculate the quantitative MR maps: T1, M0, T2 and MTR. The M0 map was non-uniformity corrected [42] for reception field inhomogeneity. Areas in the T2 map that exhibited banding artifacts (from the bSSFP acquisition) were excluded from the study. 

The 1 cm thick coronal MS brain slabs were MRI scanned to facilitate registration of histology to the high-resolution whole hemisphere MR images. The latter were first linearly registered to the coronal slabs using a 6-parameter transformation (3 rotations and 3 translations) to match the coronal plane of the histology slides. Registration of the histology slides to the MRI volume was initialized using manually defined tag points and a 12-parameter affine transformation, followed by a non-linear in-plane registration, using manually-defined masks, to correct for distortions caused by the slicing.

Regions of interest (ROIs) corresponding to CLs, NAC, WM lesions and NAWM were manually delineated on the MBP sections. The ROIs in the cortex were multiplied by an MRI mask of cortical GM that was eroded by a 3D 6-connectivity kernel to eliminate a single layer of voxels (0.35 mm isotropic) with partial volume effects with WM and cerebrospinal fluid. 

### 2.6. Statistical Analysis

Student's *t*-tests (two sided, *α* = 0.05) with Bonferroni correction for multiple comparisons were performed to compare the qMR parameters and myelin content between voxels labeled as CLs and NAC. Pearson's (*r*) and Spearman's (*ρ*) correlation coefficients were used to investigate the relationship between qMR parameters and myelin content.

## 3. Results

**Figure 2:**
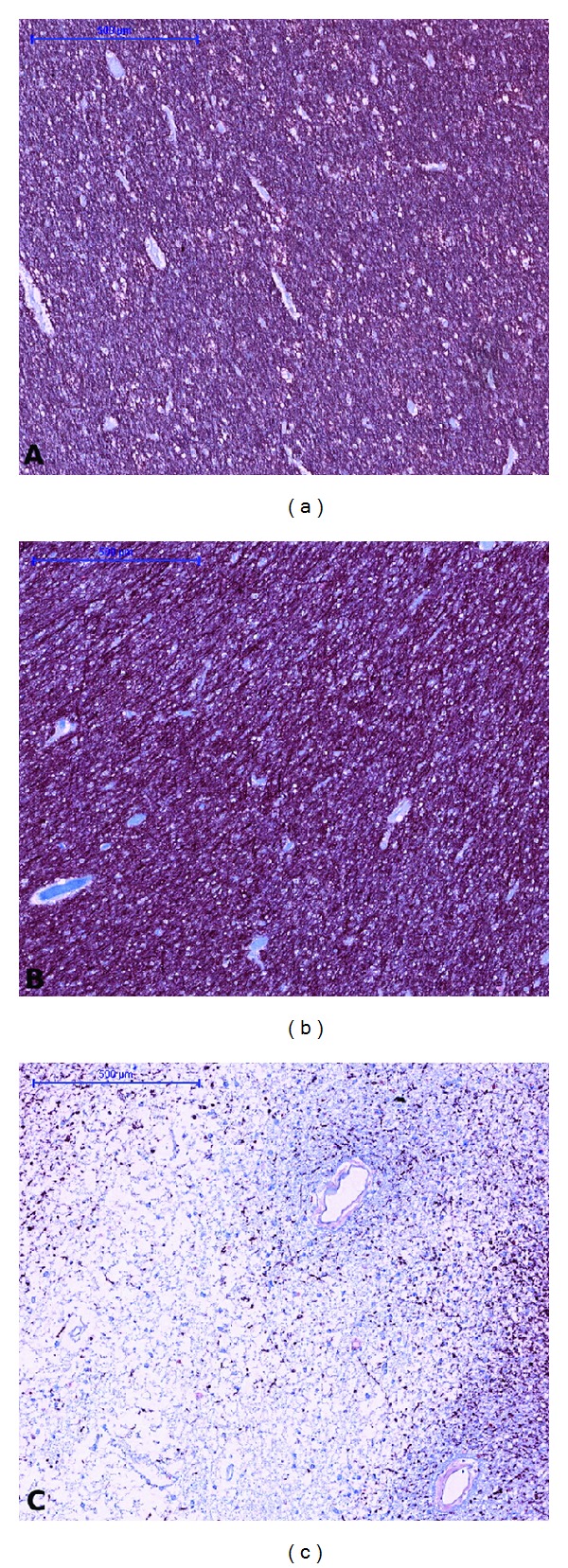
Tissue sections immuno-stained against MBP representing healthy WM (a), NAWM (b), and a demyelinated WM lesion (c). Bar = 500 *μ*m.

**Figure 3:**
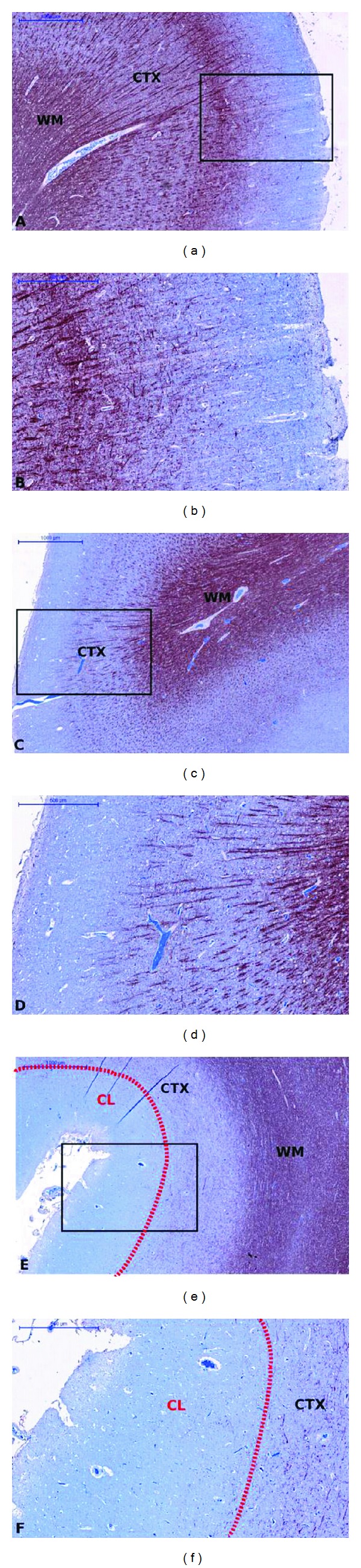
GM sections immuno-stained against MBP. The rectangles in the MBP sections in the left column are magnified in the right column. ((a)-(b)) represent healthy cortex, ((c)-(d)) NAC, and ((e)-(f)) a demyelinated type III subpial CL delineated by the dotted red line. Left column ((a), (c), (e)) bar = 1000 *μ*m, right column ((b), (d), (f)) bar = 500 *μ*m.

MBP sections of WM tissue are shown in Figure 2, where the myelin fibers are stained a dark red-brown in contrast to the light blue background from the Acid Blue 129 counterstain. The difference in myelin content between the periventricular demyelinated WM lesion Figure 2(c), healthy WM Figure 2(a) and NAWM Figure 2(b) is readily apparent. MBP stained sections of the cortex are shown in Figure 3. In the healthy cortex Figures 3(a)-3(b) and NAC Figures 3(c)-3(d), the different laminae from the myeloarchitecture, that is, the density and orientation (radial or tangential) of the myelin fibers, can be distinguished. A type III subpial lesion along the cingulate sulcus is shown in Figures 3(e)-3(f), characterized by complete demyelination of the superficial cortical layers. 

**Figure 4:**
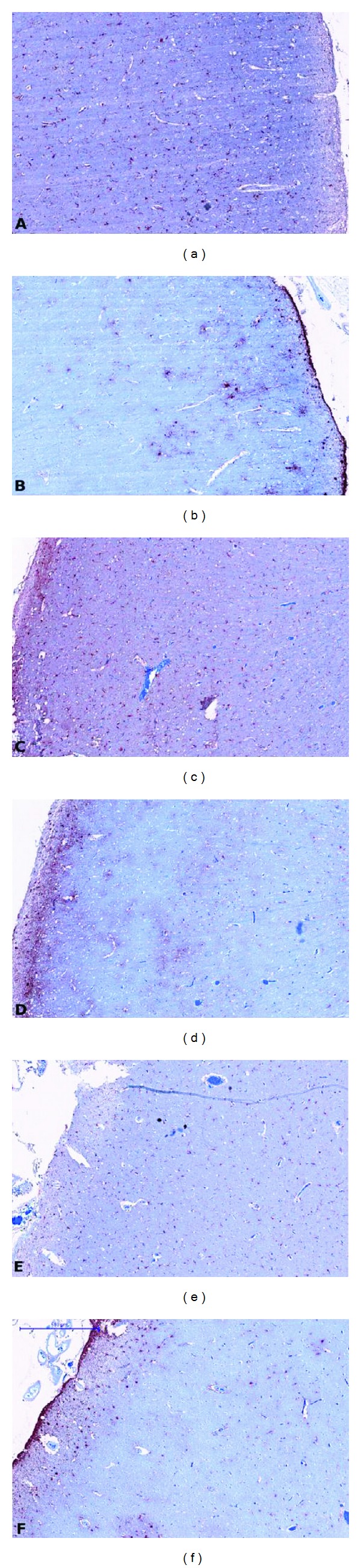
Tissue sections immuno-stained against IBA1 for macrophages and microglia (left column) and against GFAP for astrocytes (right column). ((a)-(b)) represent healthy cortex, ((c)-(d)) NAC, and ((e)-(f)) CL. Tissue sections match the MBP sections presented in Figure 3 ((b), (d), (f)). Bar = 500 *μ*m.

 The IBA1 and GFAP sections, shown in Figure 4, match the MBP sections in the second column of Figure 3. An increase in microglia was not readily visible on the IBA1 sections of CLs and NAC in comparison to healthy cortex. We observed a small increase in astrocytes, in particular in the superficial cortical layers, in GFAP sections of CLs and NAC in comparison to healthy cortex. Quantitative analysis would be required to confirm this observation. The low density of microglia and astrocytes in the cortical lesions indicates that they are chronic and inactive, which is expected given the long disease duration of this patient.

**Figure 5:**
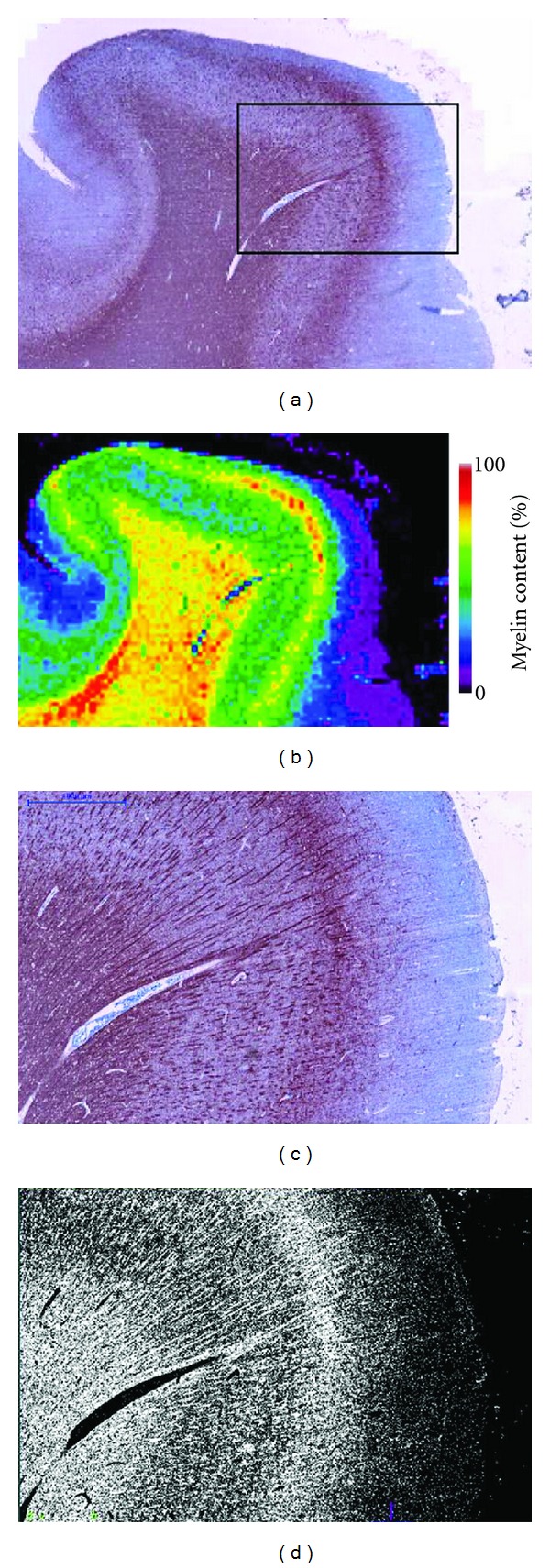
(a) MBP-stained section of healthy brain and (b) resulting myelin content map (0–100%). (c) Magnification of the region outlined by the black rectangle in the section in (b) and (d) binary classification of the myelin.

**Figure 6:**
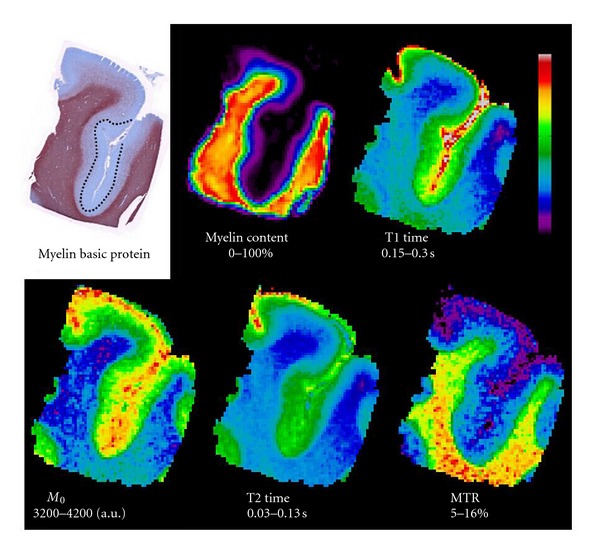
MBP stain, and co-registered myelin content and quantitative MR maps of a section of the superior frontal cortex of the MS brain with a demyelinated lesion in the fundus of the sulcus.

An example of the automated myelin fiber segmentation and resulting myelin content map for an MBP section of healthy control cortex is shown in Figure 5. An MBP section of MS cortex and corresponding co-registered myelin content and qMR maps are shown in Figure 6. 18 ROIs, including 7 in the NAC (128 mm^2^), 7 in type III subpial CLs (226 mm^2^), 2 in the NAWM (70 mm^2^) and 2 in periventricular WM lesions (27 mm^2^), were manually drawn on the MBP sections. The CLs sampled were situated in the sulci of the cingulate, insular, superior and inferior frontal, and orbito-frontal cortices.

The voxel-based qMRI and myelin content results are listed in Table 1. CLs are characterized by a statistically significant (*P* < 0.001) increase in M0, T1 and T2, and a decrease in MTR and myelin content in comparison to the NAC. The means are significantly different (*P* < 0.001) between NAWM and WM lesions as well. 

**Figure 7:**
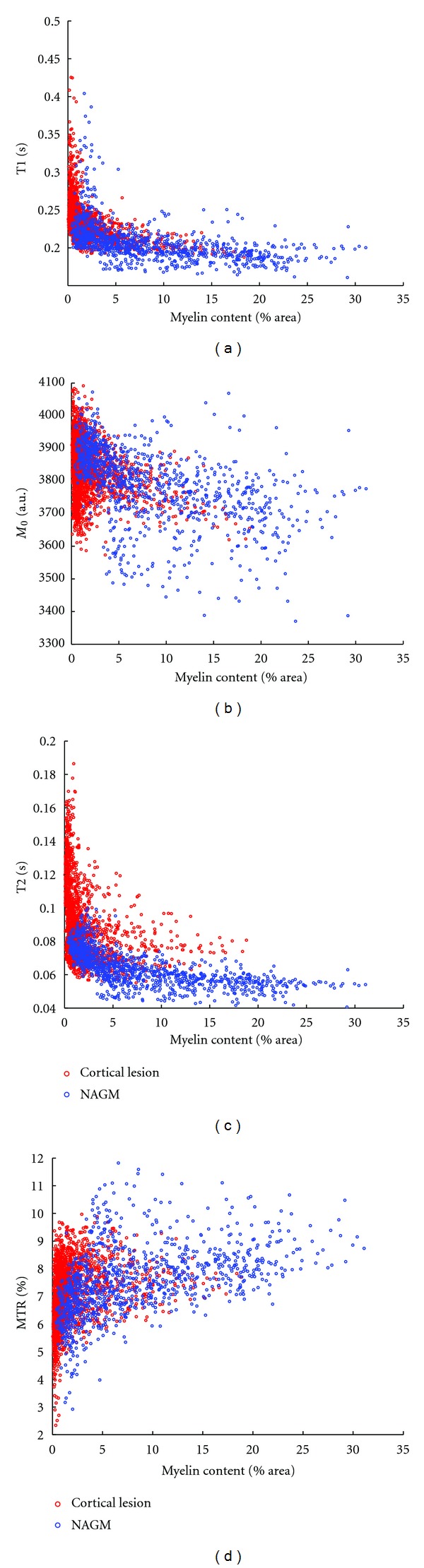
Scatter plots of the qMR parameters against myelin content. The red circles correspond to voxels labeled CL, and the blue circles to those labeled NAC.

All qMR parameters measured are correlated with myelin content: T1 (*r* = −0.58, *ρ* = −0.77, *P* < 0.001), T2 (*r* = −0.51, *ρ* = −0.65, *P* < 0.001), proton density M0 (*r* = −0.61, *ρ* = −0.59, *P* < 0.001) and MTR (*r* = 0.56, *ρ* = 0.60, *P* < 0.001). The Spearman coefficient is higher than the Pearson coefficient for both relaxation times, indicating that the relationship with myelin content may be non-linear. Scatter plots illustrating the association between the qMR parameters and myelin content are shown in Figure 7. The relaxation times increase dramatically as the myelin content tends to zero.

**Figure 8:**
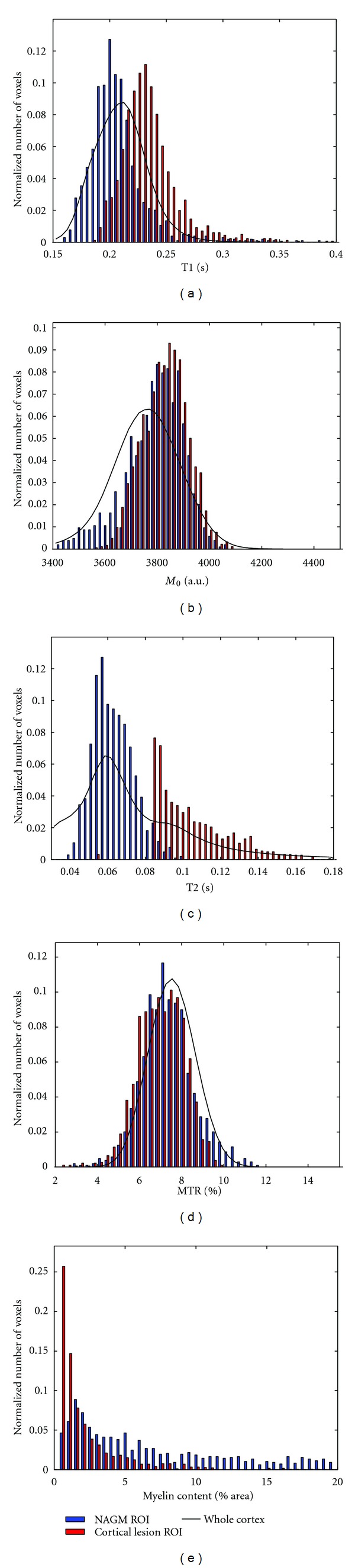
Histograms of the qMR parameters and myelin content where blue corresponds to voxels labeled NAC, red to CL, and the black line to the distribution over the entire cortex.

The histograms, normalized by the number of voxels, of the qMR parameters for CLs, NAGM and the whole cortex are shown in Figure 8. There is an extensive overlap in the distributions of the CLs and NAC for all 5 indices. Furthermore, the distributions of the qMR parameters over the whole cortex are characterized by a single peak, with the exception of T2 where a second small peak at higher times can be distinguished.

**Figure 9:**
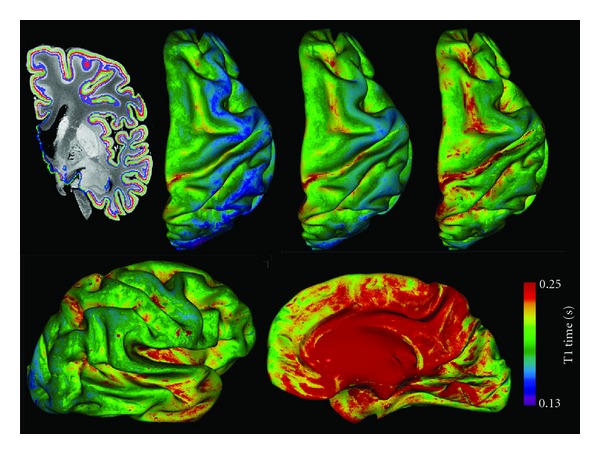
Top row: T1 times mapped onto inflated cortical contours extracted at 75%, 50% and 25% cortical depth (left to right) from the pial surface. The contours are outlined on a coronal view of an MR image in blue, red and green respectively. Bottom row: lateral and medial view of the T1 times at 25% cortical depth.

The T1 times are shown mapped onto surfaces at 75%, 50% and 25% cortical depth from the pial surface in Figure 9. Increases in T1 times are visible mainly deep in the sulci, particularly along the cingulate and insular cortices. T1 times are also longer in the more superficial layers of the cortex due to subpial cortical lesions and the natural gradient in myelin content. We can also distinguish different myeloarchitectonic areas; for instance, the decrease in T1 times at the pre- and post-central gyri and occipital pole, correspond to the myelin rich primary motor, somatosensory, and visual cortices. 

## 4. Discussion

The distribution of cortical pathology that we observed on our limited histology samples and on the cortical surfaces mapped with qMRI values are in agreement with previous observations from histology [4, 43] and MRI [25, 44, 45]. All CLs detected in the MBP sections were type III subpial lesions, the most frequent CL type and the most under-reported *in vivo*.

 In fixed tissue, demyelinated CLs are characterized by an increase in T1 and T2 relaxation times, and relative proton density M0, as well as a decrease in MTR in comparison to the NAC. The four qMR parameters were correlated with myelin content. These observations are in agreement with two recent fixed *post mortem* qMRI and quantitative neuropathology studies [18, 29]. Schmierer et al. studied fixed tissue from the motor cortex of 21 MS brains at 9.4 T. They observed a significant increase in T2 and decrease in MTR in CLs in comparison to NAC. The trend for increasing T1 times in CLs was not significant. The study by Seewann et al. looked at 16 coronal sections from 10 fixed brains with chronic MS at 1.5 T, and found significant differences between CLs and NAC in T1 and T2 times. The observed decrease in MTR was not significant. We observed a much larger increase in T2 times (42%) in comparison to Schmierer et al. (15%) and Seewann et al. (7%). Although qMR indices should, in principle, be independent of the acquisition protocol, the different T2 relaxometry techniques used could partially explain this difference. Both Seewann and Schmierer used a CMPG sequence with multiple echo times, whereas we used a bSSFP protocol. We carefully analyzed our quantitative T2 maps in order to exclude any imaging artifacts, such as off-resonance banding, and eroded our GM mask to remove any partial volume effects with cerebrospinal fluid. The three compared studies were performed at different field strengths, and the tissues had different *post mortem* delays and durations of fixation, which might also, in part, explain the differences described above. The differences may also be due to the limited number of samples in our study which do not reflect the entire spectrum of cortical MS pathology. 

 The association of myelin content with T1 times and MTR is stronger than with T2 in our study. Schmierer et al. found that T2, and to lesser degree MTR, are predictors of myelination (transmittance of MBP-stain), whereas T1 is a predictor of neuronal density. Similarly, Seewann et al. found that longer T2 times and lower MTR correlated with demyelination (transmittance of PLP-stain), and T1 times correlated with neuro-axonal density (transmittance of Nissl-stain). The non-linear relationship between relaxation times and myelin content observed here may have been concealed in the studies mentioned previously that perform region of interest based statistical comparisons, thus averaging over different cortical laminae and levels of demyelination. The non-linear image registration between MRI and histology allowed us to perform voxel-wise statistics, thus probing the relationship between qMRI parameters and pathological substrates more precisely.

 The qMR parameter distributions show that even with a high SNR and high-resolution acquisition protocol, the distributions of qMR parameters from CLs and the NAC largely overlap in fixed tissue. There is also an overlap in myelin content, hinting that the ultimate limitation to automated global intensity-driven lesion detection in MR images is not signal-to-noise ratio or resolution, but may be more related to contrast-to-noise ratio. This overlap is due to the low contrast between CLs and NAC, in particular in comparison to the variability in myelination across cortical layers, as clearly shown by the T1 times mapped onto cortical contours at different depths from the pial surface in Figure 9. The CLs detected in our tissue samples were all type III subpial lesions, affecting only the superficial cortical laminae that are less densely myelinated than the deeper laminae. The ROIs of NAC were drawn to include, as much as possible, the same laminae as the lesions for an unbiased comparison. The CNR between lesion types I, II or IV and neighbouring NAC could be stronger since they also affect the deeper layers of the cortex that are more densely myelinated. The NAC may be partially demyelinated or remyelinated in certain areas [46], further reducing the contrast. An additional source of variability in contrast-to-noise ratio is the variation in cyto- and myelo-architecture over the cortex. In this study, the tissue blocks were cut from different cytoarchitectonic regions in order to capture this variability in cortical pathology and NAC. Schmierer et al.looked exclusively at the motor cortex, yet his scatter plots between quantitative MR and histology measures also show a significant overlap between regions of interest corresponding to CLs and NAC [29].

### 4.1. Limitations

The main limitation of this study is the small sample size of cortical MS pathology. A single *post mortem* MS hemisphere was scanned and stained, and we only detected one of the four lesion types: type III subpial lesions. Although subpial lesions are the most frequent and extensive, it is important to detect the entire pathological load to study disease progression and relate it to disability. This limited sampling may contribute to the differences between the quantitative MR characteristics of cortical lesions presented here and in other studies [18, 29].

 The lesions detected were chronic and inactive, showing no inflammation on the sections immuno-stained for microglia and astrocytes. This is typical of *post mortem* tissue samples of progressive MS where the chronic lesions are less inflammatory, but show a myelin, axonal and synaptic loss [2, 4, 6, 29]. In contrast, recent biopsy studies have shown that subpial lesions in early MS are highly inflammatory, with intense myelin-laden macrophages and lymphocytic infiltrates similar to active white matter lesions [47, 48]. Longitudinal *in-vivo* MR imaging of cortical inflammation and demyelination will thus play a major role in elucidating the role of cortical lesions in the pathogenesis of MS. 

 The qMR parameters of brain tissue change dramatically following fixation [49]. The final qMR parameters vary as a function of the *post mortem* delay before fixation and the duration of fixation. Therefore, it is unsurprising that the relationship between qMR parameters and pathological substrates changes due to fixation as well. For instance, the relationship between MTR and myelin content in WM in MS is significantly weakened after fixation [50]. Schmierer et al. showed that despite significant differences in the composition of WM and GM, qMR indices change in the same direction and to a similar degree following formalin fixation [51]. We can thus expect the general trends observed in this study to remain the same *in vivo*.

### 4.2. Future Work

To expand the MR model of cortical pathology, future studies could include additional MS brain tissue samples and investigate other quantitative MR and neuropathology indices. Quantitative magnetization transfer, as opposed to semi-quantitative MTR, could improve specificity with the pathological substrates of CLs as it has in WM [50, 52]. *In vivo* MR studies have shown increases in mean diffusivity and fractional anisotropy in CLs in comparison to NAC [53], and in NAC in comparison to healthy cortex, and have suggested that this may be caused by the morphology of activated microglia [28]. As mentioned previously, T2∗-weighted images at 7 T have shown improved lesion detection and conspicuity. CLs appear as hyperintense on T2∗-weighted images and sometimes exhibit peripheral hypointense rings [25, 54], which are related to iron-rich microglia and macrophages in active and chronic active lesions [30]. This information can thus be used to stage MS lesions *in vivo* based on inflammation. 

 As an alternative to intensity-driven CL classification, our results suggest that we should consider the laminar morphology of the cortex, and how the latter is disrupted by the four types of CLs. The CL types are mainly differentiated by their morphology and location with respect to tissue boundaries. Surface-based approaches have been previously applied to increase sensitivity to cortical MS pathology in cross-sectional patient studies [45, 55]. We recently presented a CL segmentation technique using automated laminar profile-based shape analysis [56], originally applied to parcellate histological sections of the cortex [57]. The technique is demonstrated using the *post mortem* MRI-IHC data set presented here; however, it could also be applied to high resolution *in vivo* MR images [58, 59].

## 5. Conclusion

The objective of this study was to look at the MRI signature of cortical MS pathology by performing a combined *post mortem* quantitative MRI and quantitative immuno-histochemistry study. The study showed that cortical lesions are characterized by an increase in T1 and T2 relaxation times and proton density, and a decrease in MTR in comparison to the normal appearing cortex. All qMR indices were correlated with myelin content, the strongest association was with T1 times. The histogram analysis of qMR parameters and myelin content revealed that there is not a clear distinction between lesional and normal appearing cortex due to the low contrast in comparison to variations in myelination across cortical layers and areas. 

 The visualization and characterization of cortical MS pathology using MRI has improved significantly over the last decade. The continued effort to develop novel MRI acquisition and analysis techniques is necessary to capture the full extent of the disease *in vivo*, and be able to relate these findings to the underlying pathological substrates. These goals are crucial to understand the natural evolution of the disease, and to link the pathogenic mechanisms to clinical/cognitive impairment. 

The MR model of cortical MS lesions created here can help guide the design of improved MRI acquisition techniques with improved *in vivo *contrast between cortical lesions and normal appearing cortex, and in the interpretation of MR changes observed *in vivo*. This unique data set can also be used to develop image processing techniques, based on cortical and lesional morphology for instance, that could aid in the automated, unbiased detection of cortical lesions.

## Figures and Tables

**Table 1:** Mean (standard deviation) of the quantitative MR parameters and myelin content of four tissue types: normal appearing cortex (NAC), cortical lesions, normal appearing white matter (NAWM) and WM lesions. For all MR parameters and myelin content, *P*-value < 0.0001 for differences between NAC and cortical lesions, and for differences between NAWM and WM lesions.

	NAC	Cortical Lesion	NAWM	WM Lesion
T1 time (ms)	209 (27)	236 (26)	196 (18)	380 (88)
T2 time (ms)	64 (11)	91 (22)	56 (13)	121 (27)
Proton density M0 (r.u.)	3797 (114)	3835 (86)	3467 (88)	4005 (111)
Magnetization transfer ratio MTR (%)	7.47 (1.26)	7.09 (1.09)	12.82 (0.90)	6.16 (2.79)
Myelin content (% area)	7.96 (6.69)	1.96 (2.55)	74.18 (4.70)	27.55 (14.32)
